# Coherent Excitation of Optical Phonons in GaAs by Broadband Terahertz Pulses

**DOI:** 10.1038/srep38264

**Published:** 2016-12-01

**Authors:** Zhengping Fu, Masashi Yamaguchi

**Affiliations:** 1 Department of Physics, Applied Physics, and Astronomy, Rensselaer Polytechnic Institute, 110 8th Street, Troy, NY, 12180-3590 USA

## Abstract

Coherent excitation and control of lattice motion by electromagnetic radiation in optical frequency range has been reported through variety of indirect interaction mechanisms with phonon modes. However, coherent phonon excitation by direct interaction of electromagnetic radiation and nuclei has not been demonstrated experimentally in terahertz (THz) frequency range mainly due to the lack of THz emitters with broad bandwidth suitable for the purpose. We report the experimental observation of coherent phonon excitation and detection in GaAs using ultrafast THz-pump/optical-probe scheme. From the results of THz pump field dependence, pump/probe polarization dependence, and crystal orientation dependence, we attributed THz wave absorption and linear electro-optic effect to the excitation and detection mechanisms of coherent polar TO phonons. Furthermore, the carrier density dependence of the interaction of coherent phonons and free carriers is reported.

THz wave technologies have been providing a powerful tool to access and manipulate variety of material properties including molecular vibration^1^,^2^, lattice and electronic dynamics^3^,^4^,^5^, coherent antiferromagnetic spin wave^6^, and so on^7^,^8^,^9^. THz reflection and transmission spectroscopy reveals THz interaction with phonons^10^,^11^, and THz waves emitted by coherent phonons has been studied and used as THz source^12^,^13^,^14^,^15^. Excitation and control of coherent optical phonons has been studied extensively last few decades as a tool to investigate dynamical properties of materials^16^,^17^,^18^,^19^,^20^. Coherent phonons have been excited with optical laser pulses via various mechanisms including impulsive stimulated Raman scattering (ISRS)^16^,^21^,^22^, displacive excitation of coherent phonons (DECP)^23^,^24^ and screening of the surface field^18^,^25^,^26^. Due to the large energy difference between the phonons and the photons in optical frequency range, the electric field of the excitation pulse interacts with electrons, and indirectly couples to nuclei in optical excitation processes. Recently, ionic Raman scattering (IRS)^20^ using mid-infrared pulses has been demonstrated where a rectified phonon field can exert a force onto the crystal lattice and initiate coherent vibrations of atoms. In this phonon generation mechanism, photons interact with nuclei and higher energy mid-infrared phonon state plays a role of intermediate energy state of Raman process instead of electronic energy state. Direct absorption of a photon by crystal lattice is a common phonon generation mechanism in infrared spectroscopy. However, coherent excitation of optical phonons in THz frequency range by this mechanism has not been reported. The excitation of coherent optical phonons by the direct coupling of photon and phonon provides a new tool of coherent control of polar lattice motions while electronic state remains unperturbed. In addition, the types of excitable coherent phonon modes can be increased by the use of this phonon excitation mechanism. THz photon absorption follows the same selection rule as infrared spectroscopy, while ISRS follows the selection rule of Raman scattering. In case of GaAs, TO phonon excitation has not been reported by the screening of the surface field using optical pump while it can be excited by THz photon absorption as shown below. The main reason of the lack of the study on this mechanism is due to the limited bandwidths of commonly used solid state THz radiation sources such as LiNbO_3_, ZnTe, GaP^27^,^28^,^29^. In many of these THz emitter crystals, the narrower bandwidth originates from the phonon absorption within the emitter crystals. THz emitter based on laser-induced gas plasma has a broad bandwidth over 19 THz in our condition, which covers entire “THz gap”^30^,^31^,^32^. The comparison of coherent phonon excitation processes provides the opportunities to study the details of phonon interaction in condensed matter systems. THz reflection and transmission spectroscopy reveals THz interaction with phonons^10^,^11^, and THz waves emitted by coherent phonons has been studied and used as THz source^12^,^13^,^14^,^15^.

In the present work, we report the experimental study of the excitation of polar coherent optical phonons in GaAs using broadband THz pulses from laser-induced plasma in nitrogen gas. Excited coherent optical phonons were detected as reflectivity change of the optical probe pulses. The use of the optical probe allows us to monitor the phonon oscillations in the full frequency range of the bandwidth of the THz pulses without the frequency limitation by commonly used THz electro-optical sampling detectors or photoconductive antennas^33^,^34^. We observed quite different behaviors in the excitation of coherent phonons by THz pulses compare to the previously reported coherent phonon excitation by optical pulses. We show that the TO phonon excitation mechanism by THz pulses is the direct coupling of electromagnetic wave to infrared active mode unlike the case of optical excitation of coherent phonon in GaAs where plasmon-LO-phonon coupling mediates the optical excitation of phonons. Further, the effect of the carrier density is discussed.

## Results

### The scheme of THz-pump/optical-probe experiments

**Figure 1:**
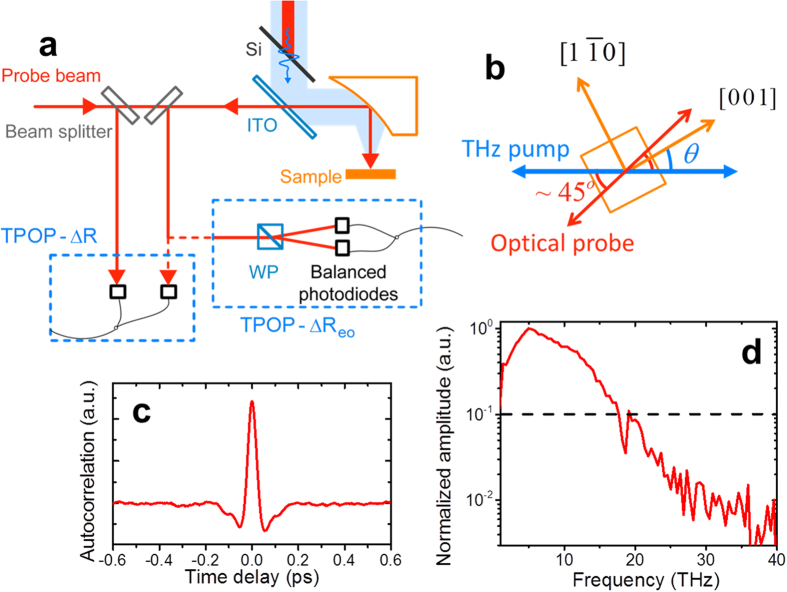
Experimental configuration and characterization of the incident THz pulses. (**a**) Experimental setup of THz-pump/optical-probe reflectivity and electro-optic reflectivity (TPOP-ΔR and TPOP-ΔR_eo_) measurement. WP is Wollaston prism and ITO is indium tin oxide glass. Balanced photodiodes are used for detection of the 800 nm optical probe. (**b**) Scheme of polarization of THz pump and optical probe in TPOP-ΔR_eo_ measurement of (110) orientated GaAs. (**c**) THz autocorrelation signal measured by THz Michelson interferometer. (**d**) Fourier spectrum of the THz autocorrelation signal. Dashed horizontal line indicates the 10% of the maximum amplitude.

The experimental setup in Fig. 1(a) was used for THz-pump/optical-probe (TPOP) measurements. Coherent optical phonons in GaAs were excited with the broadband THz pulses. Isotropic and anisotropic electro-optic reflectivity changes induced by the excited coherent phonons were probed by 800 nm optical pulses. The THz autocorrelation signal and its Fourier spectrum are shown in Fig. 1(c) and (d), respectively. Full width at half maximum of the autocorrelation signal is 38 fs and corresponding bandwidth covers the frequency region up to 19 THz with 10% or greater of the maximum Fourier amplitude. This broadband THz pulse from laser-induced plasma covers the frequency range of optical phonons in GaAs^35^, and ensure the possible excitation of the coherent lattice vibration with THz pulses.

### Transient optical reflectivity induced by THz pulses

**Figure 2:**
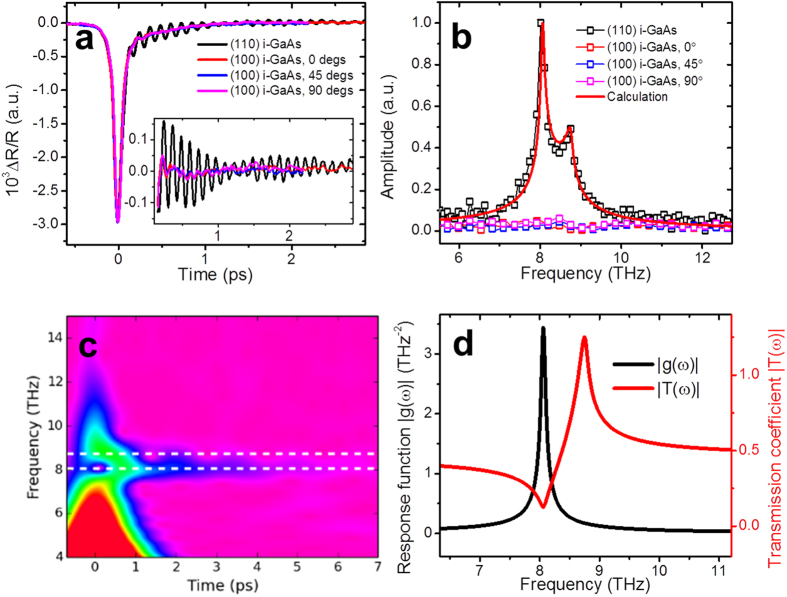
THz induced transient reflectivity change in (100) and (110) intrinsic GaAs. (**a**) TPOP-∆R signal in time domain. The labels for (100) i-GaAs indicates the angle between [011] axis and the polarization direction of the pump and probe. For the (110) i-GaAs, [1–10] crystal direction is parallel to the pump and probe polarization. The inset is the oscillatory part subtracted by the exponential background. (**b**) Fourier transform of the oscillatory signal. The calculated curve is based on equation (8) assuming impulsive time domain THz pulse and normalized to the peak at TO frequency. Slowly decaying background was subtracted prior to Fourier transformation. (**c**) The calculated curve the response function of the harmonic oscillator, *g*(*ω*) and the transmission through nitrogen/GaAs interface, *T*(*ω*).

The transient reflectivity change induced by the THz pump pulse in intrinsic (100) and (110) GaAs samples are shown in Fig. 2(a). All signals show a peak near pump-probe delay *t* = 0, and follow non-oscillatory decaying background. The background signal of the transient reflectivity relaxes to an equilibrium value within a few picoseconds regardless of the orientation of the sample, indicating the isotropic nature of the initial peak and the following decay. The possible origin of the non-oscillating background decay is energy relaxation of electrons from a non-equilibrium state to a quasi-equilibrium state in which electrons are thermalized through electron-electron interaction^36^,^37^. The behavior of this background component is quite different from the reported optically excited phonons in GaAs^17^,^18^ where the background component decays in slower time-scale of carrier recombination of nanosecond^38^,^39^,^40^. In optical pump experiment, excitation mechanism of coherent phonons is the ultrafast screening of the surface-space-charge field by the photo-excited free carriers through interband transition^17^,^18^,^23^,^25^, while no free carriers are excited by interband transition due to the smaller photon energy than the bandgap in THz pump experiment.

In Fig. 2(a), oscillation of the signal is superimposed on the decaying background component in the (110) sample while no oscillatory component is observed in the (100) sample regardless of the in-plane orientation of the crystal. The amplitude of the oscillation is modulated as seen in the inset of Fig. 2(a) rather than a monotonic decay, suggesting that more than one frequency component exists. The Fourier transform of the oscillating components is shown in Fig. 2(b). Two peaks at 8.0 ± 0.1 THz and 8.75 ± 0.1 THz are observed for undoped (110) GaAs. The frequencies of the peaks agree with the reported values of transverse optical (TO) and longitudinal optical (LO) phonon frequencies at the center of the Brillouin zone for GaAs^35^. The polarization of the THz field and longitudinal phonons with same wave vector would be orthogonal to each other. There is no sudden depolarization of the longitudinal field to initiate coherent longitudinal phonon in THz pump experiments contrary to the optical pump experiments^10^, since no free carriers are excited to screen the surface-space-charge field. Also, Berreman effect^41^ reported in thin film can be excluded as a LO phonon excitation mechanism since the pump pulse is normal incidence to the sample. Furthermore, the sample position was moved away from the focal position along the optical axis to change the ratio of longitudinal to transverse field of the THz beam^42^. However, no change of the ratio of TO and LO components of the reflection signal was observed. Therefore a possible phonon excitation by the longitudinal component of the electric field in the focus can be excluded. The origin of the peak at the longitudinal phonon frequency will be discussed in later section. No phonon peaks have been observed in (100) samples in the current experiment. In previously reported optical pump experiments, optical phonons were excited in (100) samples^17^,^18^. This is a clear indication that the optical phonon excitation mechanism by THz pulses and optical pulses are different in GaAs. Furthermore, in optical excitation experiments, only LO phonons have been excited but no TO phonons were observed in undoped GaAs^18^, while in current THz excitation experiment, TO phonons are excited. Figure 2(c) shows the wavelet chronogram of the TPOP-ΔR signal of (110) i-GaAs where horizontal lines in the figure indicate the LO and TO frequencies. The chronogram shows that the frequency component at TO frequency is missing near zero time delay, but it starts appearing near 300–400 ps. On the other hand, LO frequency component does not show such behavior. The reason of the missing TO components is not clear, however, such a behavior is not expected from the model we used in this work.

### Model of coherent phonons excited by THz pulses

Coherent phonons can be modeled using damped harmonic oscillators, and the equation of motion is given as follows^16^


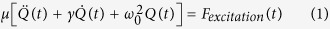


where *μ*, γ, *ω*_*o*_ and, *F*_*excitation*_(*t*) are reduced mass of the oscillator, damping coefficient, the frequency of the TO phonon mode and the driving force, respectively. In the case of the THz pump experiment of polar semiconductors such as GaAs, the lowest order of the driving force is linearly proportional to the electric field of THz pulses. Polarization *P* can be expanded in terms of electric field *E*


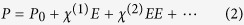


where *P*_0_ is spontaneous polarization, *χ*^(1)^ and *χ*^(2)^ are linear and second order electric susceptibilities. Each of terms in equation (2) can be expanded further in terms of the phonon coordinate of interest *Q*.


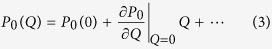



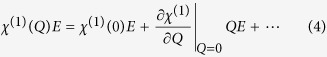


The first terms in equations (3) and (4) correspond to a spontaneous polarization when the nuclei are in their equilibrium positions and electronic contribution to the polarizability. The second terms in equation (3) and (4) correspond to the contribution to polarization from the polar phonon mode, and to the first order Raman contribution.

The driving force is given as a derivative of the interaction potential of photon and polarization, and in the lowest order of the electric field it is given as follows.


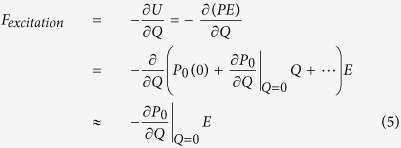


where *P*_0_(0) = 0 for GaAs. The solution of equation (1) in frequency domain is


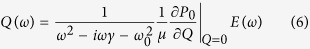


where *E*(*ω*) is the electric field inside of the sample. In order to understand the observed oscillations in the reflectivity signals at LO and TO frequencies, we need to take into account the dispersion of the transmittance near the TO and LO frequencies. The transmitted THz electric field through the gas/GaAs interface has a maximum at LO frequency where the dielectric constant becomes zero, and shows a minimum at the resonance frequency of the oscillator, i.e. TO frequency, as shown in Fig. 2(d). The driving force exerted on the crystal lattice is proportional to the local field *E*(*ω*)


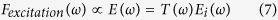


where *T*(*ω*) is transmission coefficient through the gas/GaAs interface and *E*_*i*_(*ω*) is the electric field of the incident THz pump pulse. Combining equations (6) and (7), the amplitude of excited phonon in frequency domain can be given as follows


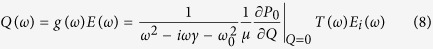


where *g*(*ω*) is the response function of the harmonic oscillator, 
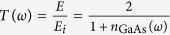
 is the frequency-dependent transmission coefficient at the gas/GaAs interface, and *n*_GaAs_(*ω*) is the frequency-dependent complex refractive index of GaAs which is modeled as damped harmonic oscillators^10^.


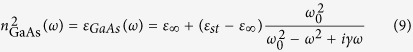


where *ε*_GaAs_(*ω*) is frequency-dependent dielectric function. *ε*_∞_ and *ε*_*st*_ are high-frequency and static dielectric constants respectively. In the case of infinitely short pump pulses, *E*_*i*_(*t*) = *δ*(*t*) and *E*_*i*_(*ω*) is constant. Figure 2(b) shows *Q*(*ω*) = *g*(*ω*)*T*(*ω*)*E*_*i*_(*ω*), and Fig. 2(d) shows the factors *g*(*ω*) and *T*(*ω*). Although this model assume only one resonance at TO frequency, the calculated spectrum of *Q* shows peaks at TO and LO frequencies, and agrees with the experimental data as seen in Fig. 2(b). *T*(*ω*) shows the maximum transmission at LO frequency. Transverse oscillation at LO frequency is due to the THz field at LO frequency, drives the harmonic oscillator with resonance at TO frequency, off resonantly.

### Pump/probe polarization dependence and pump-power dependence

**Figure 3:**
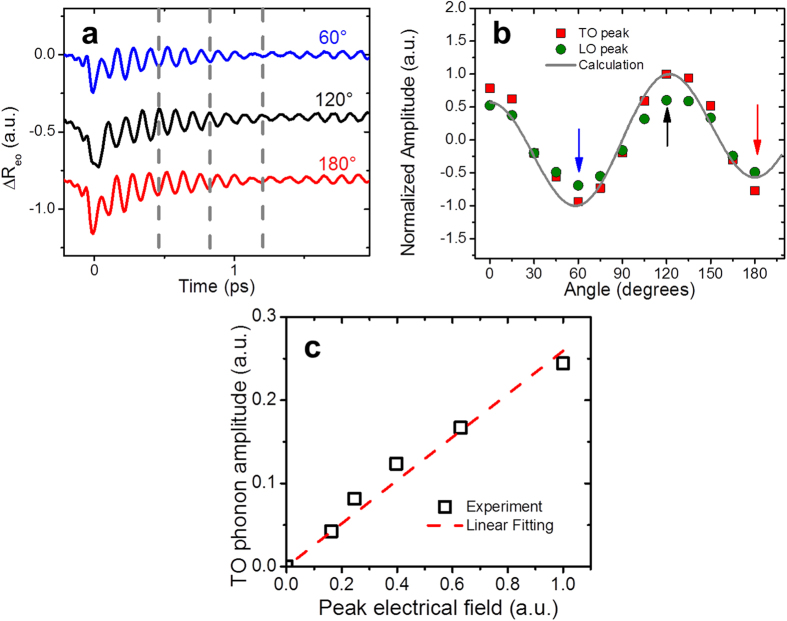
The pump/probe polarization dependence and pump field dependence of TPOP-∆R_eo_ signals in (110) undoped GaAs. (**a**) Time-domain signals at the crystal rotation angle 60/120/180 degrees equivalent to different pump/probe polarizations. (**b**) The pump/probe polarization dependence of the Fourier component at TO and LO frequencies. The amplitudes are normalized to the maximum points near 120 degrees. The temporal signals correspond to sample rotation angles 60/120/180 degrees, as indicated by black/red/blue arrows in (**a**) respectively. (**c**) Dependence of coherent TO phonons amplitude on the peak electric field of the THz pump.

To understand the nature of the observed oscillatory components further, we measured transient electro-optic reflectivity change, ∆R_eo_ in which isotropic contributions are eliminated^17^,^19^. The polarization direction of the pump and probe pulses are set to 45° away from each other, and the sample was rotated about (110) axis (Fig. 1(b)). Therefore the polarizations of the pump and probe changes relative to the crystal axes. Figure 3(a) shows the transient electro-optic reflectivity change in three different pump/probe polarizations. The phase of the oscillations changes 180° every 60° of the sample rotation. The pump/probe polarization dependence of the amplitude is shown in Fig. 3(b). The solid line was calculated assuming the linear electro-optic effect, and agrees with the experimental data. In zincblende structure, there are three non-zero Pockels tensor components and all of these have the same value^43^. ΔR_eo_ in the current experimental configuration is induced by the lattice displacement *Q* which has the same polarization direction as the pump THz field. The calculated pump/probe polarization dependence of ΔR_eo_ based on the linear electro-optic effect agrees with the experimental data (Fig. 3(b)). Furthermore, the model predicts that the transverse field induces the birefringence in (110) plane, but not in (100) plane. Our experimental results agree with the model as seen in Fig. 2(b), suggesting both TO and LO oscillatory components share the same polarization. Further, when only the probe polarization is rotated, both oscillation contributions disappear simultaneously at a same rotation angle. Although our model includes only one harmonic oscillator at TO frequency, the spectrum shows the second peak at LO frequency. The LO peak is due to a harmonic oscillator with resonance at TO frequency is driven by an external force with off-resonant frequency LO frequency. It has a peak at LO frequency because of the transmittance has a maximum at the frequency. The ions move in the direction of the driving force of the transverse electric field of the incident THz pulse. Quantitatively the lattice displacement is given by the product of the response function and the transmitted electric field spectrum which has the maximum at the LO frequency (equation (8)). Figure 3(c) shows the pump field dependence of the TO phonon amplitude. The linear dependence of the phonon amplitude on the pump field supports the THz wave absorption as the driving force of phonon generation. The result shows the contrast to the optical pump experiment where the driving force is proportional to intensity of the pump pulse for all following phonon generation mechanisms such as DECP^23^, ISRS^16^ and screening of built-in field^25^. Considering the pump/probe polarization dependence, the pump field dependence, and the fact that the Fourier spectrum can be reproduced by the product of the transmittance and response function, the oscillation component observed at LO frequency should not be the LO phonons but the contribution from the transmitted pump electric field, which forms phonon-polariton in the sample.

**Figure 4:**
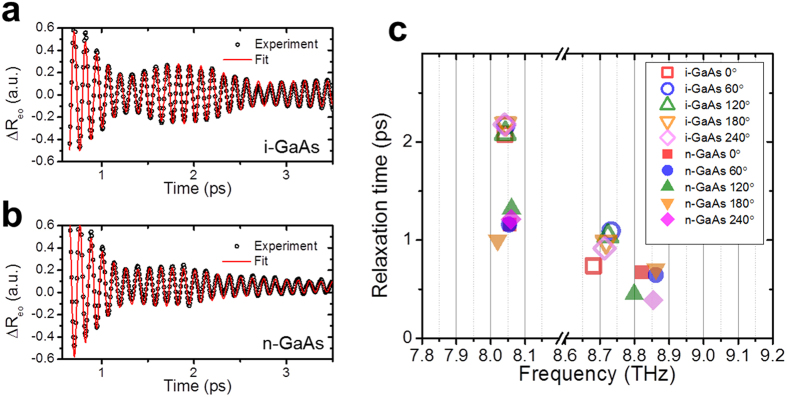
THz-excited cohernent phonons in doped and undoped GaAs. (**a**) and (**b**) are TPOP-ΔR_eo_ signals and fitting in time domain for undoped and doped (110) GaAs respectively. (**c**) Shows the relaxation times and oscillation frequencies obtained by fitting the time-domain signals measured at different angles of the samples.

**Figure 5:**
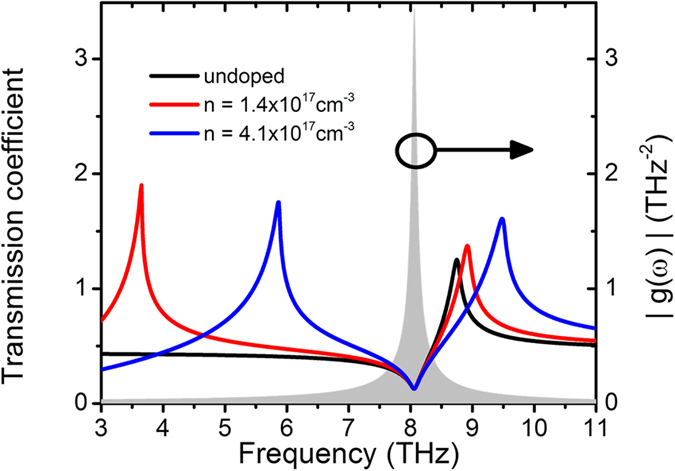
Frequency-dependent transmission coefficients of GaAs with different carrier densities. Black/red/blue curves are transmission coefficients *T*(*ω*) of gas/GaAs interface for different carrier densities of GaAs. Grey part indicates the response function of the harmonic oscillators *g*(*ω*).

The effect of carrier doping on the THz-excited coherent phonons is shown in Fig. 4 by comparing intrinsic and n-doped (110) GaAs. The TPOP-∆R_eo_ signals were fitted with the following phenomenological function:


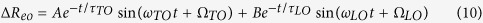


where *ω*_*TO*_, *ω*_*LO*_, *τ*_*TO*_, *τ*_*LO*_, Ω_*TO*_ and Ω_*LO*_ are resonant frequencies, relaxation times, and initial phase of the oscillations at TO and LO frequencies, respectively. Equation (10) assumes two independent oscillators were used to represent the experimental signals phenomenologically to evaluate the changes on the frequency and the damping due to the carrier doping, however, it is not our intention to suggest that the oscillator at LO frequency is due to LO phonon. The fit for intrinsic and n-doped samples are shown in Fig. 4(a) and (b). The fitted relaxation time and frequencies are summarized in Fig. 4(c). Stronger damping constants were observed for both of oscillations at TO and LO frequencies in doped sample. The TO frequency shows no change, while peak at LO frequency shows blue shift about 0.1 THz in doped samples. The latter is attributed to the change of dielectric function due to the increased free carrier contribution. The free carrier contribution to the dielectric function was taken into account by Drude model, which was added to equation (9). The increased free carrier density shifts the maximum of the transmission spectrum which determines the higher frequency peak position of ΔR and ΔR_eo_ signal as shown in Fig. 5. On the other hand, the TO frequency is not affected by free carrier contribution^44^ as it is determined by the resonant frequency of the oscillators. The experimental results are reproduced well with the model. The peak at LO frequency should be distinguished from LO-plasmon coupled mode^17^,^44^,^45^,^46^ since the polarization of the observed mode at LO frequency is perpendicular to the direction of the incidence and has transverse electric field, rather than longitudinal direction. The interactions of free carriers and lattice are not considered in our model, hence, LO-plasmon coupled mode does not appears in our calculation. However, the frequency shift with carrier doping is explained within in our model.

The relaxation time of the THz-excited coherent TO phonons *τ*_*TO*_, is 2.1 ps for the undoped sample and 1.2 ps for the n-type sample. The former is close to the dephasing time of TO phonons measured by coherent anti-Stokes Raman scattering (CARS) technique^47^, ~2.8 ps. The CARS experiment also shows that the presence of a moderate density electron plasma (2 × 10^17^ cm^−3^) does not change the TO dephasing time significantly^47^. On the other hand, the relaxation time of the oscillation near LO frequency, was ~1 ps, which is only about a half of the reported dephasing time of coherent LO phonon in intrinsic GaAs^18^, ~2 ps, which supports that the oscillatory signal we observed in our experiment is not coherent LO phonons.

## Conclusion

In conclusion, we have demonstrated coherent phonon excitation using broadband THz pump pulses in GaAs. TPOP-∆R and TPOP-∆R_eo_ experiments were used to investigate the coherent phonon excitation and detection. The linear dependence of the phonon amplitude on the pump electric field indicates the direct coupling between the THz pump electric field and dipole moment carried by polar phonons as the generation mechanism for TO phonons. The oscillatory component at LO frequency is not longitudinal phonon mode, but caused from the THz pump pulse transmitted through the gas/GaAs interface, which forms the phonon-polariton. For the first time, THz-excited coherent TO phonons in GaAs were observed. We also determined the relaxation times of coherent TO phonon with carrier doping and verified that the relaxation time of coherent TO phonons decreases with higher carrier concentration.

## Methods

The experimental setup in Fig. 1(a) was used for TPOP measurements. Optical pulses from a Ti-sapphire laser with a center wavelength at 800 nm were used as a light source. The energy and duration of pulses are 4 mJ/pulse and 35 fs respectively. The pulse repetition rate was 3 kHz. Broadband THz pulses were generated using two-color excitation scheme in laser induced nitrogen gas plasma^30^,^31^,^32^. The second harmonic pulses were generated by using a type I beta barium borate (BBO) crystal, and both of the fundamental and second harmonic pulses were focused into nitrogen gas to generate THz waves. THz Michelson interferometer^48^ were used to characterize the THz pulse width and spectrum. The THz pump and optical probe pulses were combined using an ITO glass plate and focused with an off-axis parabolic mirror. Both pulses were directed to the sample at the normal incidence. Transient optical reflectivity change (∆R) and electro-optic reflectivity change (∆R_eo_)^17^,^49^ were measured in two experimental configurations. In ∆R_eo_ measurement, a Wollaston prism was used to separate horizontally and vertically polarized components of the reflected probe beam. The polarization of THz beam was dominantly horizontal, the same as the polarization of optical pump used to excite plasma^32^. The polarization of the optical probe was set to the horizontal in ∆R measurement, while 45 degrees from the horizontal direction in ∆R_eo_ measurement (Fig. 1(b)). All THz beam path was enclosed in a chamber purged with dry nitrogen to minimize the absorption of water vapor. High resistivity silicon wafers (>10,000 Ω.cm) were used to attenuate the THz pump pulse for the pump power-dependent measurements. We did not observed any spectral shape change due to the attenuation except the phonon absorption peak of Si substrate around 17 THz. Three kinds of VGF-grown (vertical gradient freeze method) GaAs wafers were used in experiments: Undoped (100) and (110), and Si-doped n-type (110) GaAs wafers. The free carrier concentration of the n-type GaAs is (1.4–4.1) × 10^17^/cm^3^. For the in-plane orientation dependence of the (100) crystal, the angle *θ* was measured from the direction of the pump polarization to (010) direction, and for (110) sample, *θ* was measured from (001) direction.

## Additional Information

**How to cite this article**: Fu, Z. and Yamaguchi, M. Coherent Excitation of Optical Phonons in GaAs by Broadband Terahertz Pulses. *Sci. Rep.* **6**, 38264; doi: 10.1038/srep38264 (2016).

**Publisher’s note:** Springer Nature remains neutral with regard to jurisdictional claims in published maps and institutional affiliations.
